# The *mir-51* Family of microRNAs Functions in Diverse Regulatory Pathways in *Caenorhabditis elegans*

**DOI:** 10.1371/journal.pone.0037185

**Published:** 2012-05-16

**Authors:** John L. Brenner, Benedict J. Kemp, Allison L. Abbott

**Affiliations:** Department of Biological Sciences, Marquette University, Milwaukee, Wisconsin, United States of America; Brown University, United States of America

## Abstract

The *mir-51* family of microRNAs (miRNAs) in *C. elegans* are part of the deeply conserved miR-99/100 family. While loss of all six family members (*mir-51-56)* in *C. elegans* results in embryonic lethality, loss of individual *mir-51* family members results in a suppression of retarded developmental timing defects associated with the loss of *alg-1*. The mechanism of this suppression of developmental timing defects is unknown. To address this, we characterized the function of the *mir-51* family in the developmental timing pathway. We performed genetic analysis and determined that *mir-51* family members regulate the developmental timing pathway in the L2 stage upstream of *hbl-1*. Loss of the *mir-51* family member, *mir-52*, suppressed retarded developmental timing defects associated with the loss of *let-7* family members and *lin-46*. Enhancement of precocious defects was observed for mutations in *lin-14, hbl-1,* and *mir-48(ve33)*, but not later acting developmental timing genes. Interestingly, *mir-51* family members showed genetic interactions with additional miRNA-regulated pathways, which are regulated by the *let-7* and *mir-35* family miRNAs, lsy-6, miR-240/786, and miR-1. Loss of *mir-52* likely does not suppress miRNA-regulated pathways through an increase in miRNA biogenesis or miRNA activity. We found no increase in the levels of four mature miRNAs, let-7, miR-58, miR-62 or miR-244, in *mir-52* or *mir-52/53/54/55/56* mutant worms. In addition, we observed no increase in the activity of ectopic lsy-6 in the repression of a downstream target in uterine cells in worms that lack *mir-52*. We propose that the *mir-51* family functions broadly through the regulation of multiple targets, which have not yet been identified, in diverse regulatory pathways in *C. elegans*.

## Introduction

microRNAs (miRNAs) are ∼22 nucleotide, non-coding RNAs that post-transcriptionally regulate the expression of their downstream targets. miRNAs bind to sites with imperfect complementarity in target mRNAs [1], which, in most cases, results in lower target protein levels due to the inhibition of translation and the reduced stability of target mRNAs [2], [3]. The effects of miRNA regulation on target protein levels can vary widely. In some cases, miRNA binding to a target can function as a ‘switch’ by directing the nearly complete suppression of target mRNA translation. For example, the lin-4 and let-7 miRNAs function as developmental switches to strongly down-regulate their respective targets, *lin-14* and *lin-41* [4]-[7]. In other cases, miRNA binding to a target can function as a ‘fine tuner’ to direct modest repression of target mRNA translation. For example, in flies, miR-8 maintains the levels of *atrophin* in an optimal range [8]. However, in recent work, Mukherji et al. [9] demonstrate that the effect of miRNA regulation upon target protein levels is not an inherent property of the miRNA but rather depends on the stoichiometry and binding affinity of a miRNA and its associated target mRNAs. At low levels of target mRNA, a miRNA can act to strongly repress translation, whereas at high levels of the target mRNA, a miRNA can act to modestly repress translation [9].

While penetrant mutant phenotypes are observed in *lin-4* and *let-7* mutants, defects were not identified for most individual miRNA mutants in *C. elegans* [10], though progress in identifying functions for miRNAs is being made [11]. For some miRNA mutants, like *let-7* or *mir-35* family mutants, the lack of observed defects is a result of functional redundancy among miRNA family members, which share a six nucleotide 5′ seed sequence [12], [13]. For other miRNA mutants, like *lsy-6*, the lack of obvious defects reflects highly specialized functions for individual miRNAs that were not observed in broad-based phenotypic analyses [14]. Furthermore, since some miRNAs function to modestly regulate, or fine tune, target gene expression, the loss of these miRNAs may not result in obvious defects during normal growth conditions. Approaches that have examined miRNA mutant worms under conditions of stress, such as altered environmental conditions [15], [16] or genetic backgrounds [17], have been successful in identifying mutant phenotypes associated with the loss of individual miRNAs.

Using a sensitized genetic background, we characterized defects in 80% of the individual miRNA mutants analyzed [17]. For that analysis, we used strains that lack one of two Argonaute proteins that function in the miRNA pathway in *C. elegans*, ALG-1, as a sensitized background in which to identify mutant phenotypes. In *alg-1* mutants, overall miRNA levels are reduced, including the lin-4 and let-7 miRNAs, which leads to observable defects in the developmental timing pathway [18]. This pathway controls the appropriate temporal execution of stage-specific developmental programs through the four larval stages, L1–L4 [19]. Loss of *alg-1* activity results in developmental timing defects including incomplete alae formation at the L4 to adult transition, an increased number of hypodermal seam cells, and a failure to exit the molting cycle [17], [18], [20]–[23]. Loss of *mir-51* family members partially suppresses these developmental timing defects in *alg-1* worms [17].

The *mir-51* family is part of the larger miR-99/100 family, a miRNA family that shows deep conservation from cnidarians through humans [24]. In *C. elegans*, the *mir-51* family comprises six miRNAs, miR-51 through miR-56. Loss of the entire *mir-51* family in *C. elegans* results in embryonic lethality, due to a failure of pharyngeal attachment [25]. Loss of multiple members causes several mutant phenotypes including larval lethality and slow growth [13], [25]. These pleiotropic phenotypes indicate that *mir-51* family members likely function to regulate multiple downstream targets and pathways. The mechanism whereby loss of individual *mir-51* family members suppresses *alg-1* developmental timing defects is unclear. Unlike other genes that regulate developmental timing, *mir-51* family members are expressed broadly and abundantly throughout the life of the worm [25]–[29]. We therefore wanted to determine the function of the *mir-51* family members in the regulation of the developmental timing pathway.

Here, we have defined the genetic interactions of *mir-51* family members with components of the developmental timing pathway. Additionally, we report that the *mir-51* family interacts with multiple, diverse, miRNA regulated genetic pathways, including pathways regulated by the *let-7* and *mir-35* family miRNAs, as well as lsy-6, miR-240/786, and miR-1. We provide evidence that is inconsistent with the model that the *mir-51* family regulates miRNA biogenesis or miRNA activity. Instead, we propose that the *mir-51* family functions to regulate multiple targets in diverse developmental pathways in *C. elegans*.

## Results

### Loss of *mir-51* family members partially suppresses retarded developmental timing phenotypes

The loss of *mir-51* family members suppresses *alg-1* developmental timing defects [17], suggesting a possible direct role in the regulation of the developmental timing pathway. However, mutants lacking individual *mir-51* family members did not display developmental timing abnormalities such as defects in alae formation or defects in seam cell divisions (Table 1 and Table 2). Further, worms that are multiply mutant for 5 out of 6 members of the *mir-51* family, *mir-52/53/54/55/56*, also do not display alae formation defects (Table 1 and Table 2), despite displaying other mutant phenotypes including slow growth and larval lethality [13], [25]. Because the *alg-1* developmental timing defects are similar to those associated with the loss of the *let-7* family miRNAs [18], we determined if loss of individual *mir-51* family members was sufficient to suppress *let-7* timing defects. To do this, we used a temperature sensitive *let-7* allele, *n2853*. At 25°C, these *let-7(ts)* mutants display a repetition of a late larval program with failure to form complete alae and lethality due to bursting at the vulva at the L4 to adult transition ([5]; Table 1). Loss of *mir-51* family members did not suppress either of these phenotypes in *let-7* mutants (Table 1).

**Table 1:** Genetic interactions of the *mir-51* family with retarded developmental timing mutants.

		Alae at L4 to Adult transition				Lethality		
Strain^a^	seam cells^b^	complete	gapped	none	n	% burst	% bag of worms	n
RG733 *wild type*	16.0	100	0	0	20	0	0	208
RF481 *wild type*	16.1	100	0	0	20	0	0	109
RF491 *mir-51*	16.2	100	0	0	20	0	0	151
RF499 *mir-52*	15.9	100	0	0	20	0	0	181
RF483 *mir-53*	16.1	100	0	0	20	0	0	176
RF399 *mir-54/55/56*	16.1	99	1	0	98	0	0	228
RF692 *mir-52/53/54/55/56*	–c	100	0	0	16	–	–	–
MT7626 *let-7ts* @25°C	–	0	50^d^	50	16	100	–	103
RF447 *mir-51; let-7ts* @25°	–	0	80^d^	20	20	100	-	119
RF448 *mir-52; let-7ts* @25°	–	7	73^d^	20	15	96	–	114
RF449 *mir-53; let-7ts* @25°	–	0	53^d^	47	17	99	1	92
RF442 *mir-54/55/56; let-7ts* @25°	–	7	21^d^	71	14	99	1	91
RF554 *mir-48/84/241*	22.6	0	100	0	40	56	37	111
RF556 *mir-52; mir-48/84/241*	17.7^e^	49^f^	51	0	39	3^f^	77	90
RF553 *mir-48/84/241*	22.7	0	100	0	37	66	26	125
RF555 *mir-51; mir-48/84/241*	21.8	0	100	0	37	42^g^	41	112
RF557 *mir-53; mir-48/84/241*	22.2	0	100	0	38	49^g^	39	134
RF558 *mir-54/55/56; mir-48/84/241*	20.6^h^	21^g^	79	0	38	25^g^	57	141
VT1064 *mir-48/84*	–	–	–	–	–	0	69	236
RF451 *mir-51; mir-48/84*	–	–	–	–	–	0	30^i^	101
RF469 *mir-52; mir-48/84*	–	–	–	–	–	0	5^i^	148
RF454 *mir-53; mir-48/84*	–	–	–	–	–	0	62	106
RF415 *mir-54/55/56; mir- 48/84*	–	–	–	–	–	0	2^i^	93
RF619 *mir-48/241*	19.1	5	95	0	21	31	49	144
RF730 *mir-48/241; mjEx160[mir-54/55/56]*	22.1^j^	9	91	0	32	66^k^	24^k^	136^k^
RF568 *lin-46 @15*°	19.4	5	95	0	40	–	–	–
RF569 *mir-52; lin-46 @15*°	17.8^l^	23^m^	77	0	39	–	–	–
VC894 *puf-9*	–	29	71	0	34	–	–	–
RF578 *mir-52; puf-9*	–	34	66	0	50	–	–	–
RF620 *mir-52; mir-48/241*	16.6^n^	85	15	0	20	–	–	–
RF625 *mir-48/241; puf-9*	19.2	0	100	0	19	–	–	–
RF626 *mir-52; mir-48/241; puf-9*	16.2^o^	0	100	0	17	–	–	–

a Full genotype information can be found in Table S1.

b seam cells counted in L4-stage worms using *wIs78* or *wIs79[scm::gfp]*, n≥18 (range 19–30).

c indicates results not determined.

d alae scored categorized as partially or faintly visible rather than gapped as elsewhere.

e indicates significant difference compared to RF554 *mir-48/84/241* (student's t-test, p<0.05), which contained wIs79.

f indicates significant difference compared to RF554 *mir-48/84/241* (χ2, p<0.05) which contained wIs79.

g indicates significant difference compared to RF553 *mir-48/84/241* (χ2, p<0.05) which contained wIs78.

h indicates significant difference compared to RF553 *mir-48/84/241* (student's t-test, p<0.05), which contained wIs78.

i indicates significant difference compared to VT1064 *mir-48/84* (χ2, p<0.05).

j indicates significant difference comparing worms from the same strain, but +/− for *mjEx160[mir-54/55/56]* (student's t-test, p<0.05).

k population scored for lethality is a mix of worms +/− for *mjEx160[mir-54/55/56]*.

l indicates significant difference compared to RF568 *lin-46* (student's t-test, p<0.05).

m indicates significant difference compared to RF568 *lin-46* (χ2, p<0.05).

n indicates significant difference compared to RF619 *mir-48/241* (student's t-test, p<0.05).

o indicates significant difference compared to RF625 *mir-48/241; puf-9* (student's t-test, p<0.05).

The *let-7* family members, *mir-48*, *mir-84*, and *mir-241*, function together to control the timing of the L3 stage program through down-regulation of their target, *hbl-1* [12]. In the L2 stage, a subset of hypodermal seam cells undergo two rounds of cell division resulting in an increase in the number of seam cells from 10 to 16. In mutants lacking *mir-48*, *mir-84* and *mir-241* (hereafter referred to as *mir-48/84/241)*, the L3 stage program is not executed properly and the L2 stage program is reiterated. This reiteration of the L2 stage program results in an increased number of seam cells [12]. *mir-48/84/241* mutant worms often display defects in alae formation at the L4 to adult transition. In addition, many of these mutants burst at the L4 to adult transition or execute an extra adult-stage molt, which leads to the “bag-of-worms” phenotype [12]. *mir-52; mir-48/84/241* had fewer seam cells than *mir-48/84/241* worms, indicating a suppression of the L2 reiteration phenotype (Table 1). Additionally, loss of *mir-52* suppressed the alae formation defects and bursting phenotypes of *mir-48/84/241:* 100% of *mir-48/84/241* displayed incomplete alae formation and 56% of *mir-48/84/241* worms burst at the L4 to adult transition compared to 51% and 3% of *mir-52;mir-48/84/241* mutants, respectively (Table 1). However, 77% of *mir-52; mir-48/84/241* worms showed the bag of worms phenotype, indicating an extra adult-stage molt. This likely reflects a partial suppression of *mir-48/84/241* phenotypes, rather than an inability to suppress molting since loss of *mir-52* strongly suppresses the ectopic molting phenotype of *alg-1* worms [17] as well as *mir-48/84* double mutant worms (Table 1).

Next, we examined the effect of elevated expression of *mir-51* family members on the retarded development of *mir-48 mir-241* (*mir-48/241*) mutant worms. To accomplish this, we used *mjEx160*, an extrachromosomal array with the genomic fragment for *mir-54/55/56* that was previously shown to rescue the embryonic lethality of *mir-51* family mutant worms [25] and the developmental timing phenotypes in *mir-54/55/56 alg-1* mutant worms [17]. *mjEx160* enhanced developmental timing defects of *mir-48/241* mutant worms (Table 1). *mir-48/241* worms have 19.1 seam cells on average. This is increased to 22.1 in *mir-48/241; mjEx160* worms (Table 1). This indicates elevated expression of *mir-51* family members enhances the L2 repetition phenotype.

We determined if the loss of *mir-51* family members can suppress the phenotypes of *lin-46* and *puf-9*, mutants that display retarded developmental timing defects [30], [31]. *lin-46* functions in parallel to the *let-7* family to control the timing of the L3 stage program [12], [30]. *lin-46* mutants fail to properly execute the L3 stage program and show reiteration of the L2 program at 15°C. *lin-46* mutants display extra seam cells and incomplete alae formation [30]. Loss of *mir-52* partially suppressed *lin-46* developmental timing defects: *mir-52; lin-46* double mutant worms had fewer seam cells and displayed weaker alae defects compared to *lin-46* mutant worms (Table 1). Loss of the other *mir-51* family members had no significant effect on *lin-46* developmental timing defects (data not shown). *puf-9* encodes a *pumilio* family protein that acts to negatively regulate *hbl-1* through its 3′UTR [31]. *puf-9* mutant worms fail to form complete alae at the L4 to adult transition. Loss of *mir-52* did not suppress the *puf-9* alae defects (Table 1). This suggests that *puf-9* may function downstream of the *mir-51* family to regulate developmental timing.

To determine if *puf-9* is necessary for *mir-52*-mediated suppression of the *let-7* family developmental timing defects, we examined worms multiply mutant for *mir-52*, *puf-9*, and *let-7* family miRNAs, *mir-48* and *mir-241* (*mir-48/241*). Loss of *mir-52* suppressed the seam cell and alae formation defects in *mir-48/241* mutants (Table 1). Loss of *puf-9* did not affect the *mir-52* mediated suppression of the extra seam cell phenotype of *mir-48/241* mutant worms (Table 1). However, no suppression of alae formation defects was observed in *mir-52; mir-48/241; puf-9* worms relative to *mir-52; mir-48/241* (Table 1). This is consistent with a function for *puf-9* later in development, after the L2 to L3 transition. Together, these data indicate that the *mir-51* family functions to regulate the execution of the L3 stage program, acting either downstream or in parallel to the *let-7* family miRNAs and *lin-46* and may have additional activity in the control of alae formation in late larval development.

**Table 2:** Genetic interactions of *mir-51* family with precocious developmental timing mutants.

	Precocious Alae^b^			
Strain^a^	complete	gapped	none	n
RG733 *wild type*	0	0	100	9
RF481 *wild type*	0	0	100	12
RF491 *mir-51*	0	0	100	14
RF499 *mir-52*	0	0	100	13
RF483 *mir-53*	0	0	100	15
RF399 *mir-54/55/56*	0	0	100	13
RF692 *mir- 52/53/54/55/56*	0	0	100	15
RG490 *mir-48(ve33)*	0	55	45	47
RF583 *mir-52; mir-48(ve33)*	0	88^d^	12	34
RF534 *hbl-1*	0	76	24	41
RF535 *mir-52; hbl-1*	2^d^	95	2	44
RF563 *lin-14ts @25°C*	34	66	0	29
RF588 *mir-52; lin-14ts @25°C*	76^d^	20	4	25
RF536 *lin-41*	0	11	89	38
RF537 *mir-52; lin-41*	0	14	86	36
RF538 *lin-42*	0	89	11	37
RF541 *mir-52; lin-42*	3	93	3	29
VT517 *lin-28* c	5	90	5	20
RF573 *mir-52; lin-28* c	0	100	0	20

a full genotype information can be found in Table S1.

b alae were scored in L3 molt or early L4-stage worms, except where otherwise noted.

c alae were scored in the L2 molt.

d indicates significant difference between strains of same genotype +/− *mir-52* (χ2, p<0.05).

### Loss of *mir-52* enhances precocious developmental timing phenotypes

We next characterized genetic interactions between *mir-52* and a set of precocious developmental timing genes. Loss of *mir-52* enhanced the developmental timing defects observed in three precocious mutants: *mir-48(ve33), hbl-1(ve18)*, and *lin-14(n179)*. (Table 2). First, *mir-48*(*ve33)* worms display early accumulation of miR-48 and precocious formation of adult-specific alae one stage early at the L3 to L4 transition [32]. Loss of *mir-52* significantly enhanced this precocious alae formation in the *mir-48(ve33)* background (Table 2). We found that 55% of *mir-48(ve33)* mutants displayed precocious alae formation compared to 88% of *mir-52;mir-48(ve33)* worms. Next, *hbl-1* is a central regulator of the L2 versus L3 cell fate decision [33], [34]. Loss of *mir-52* enhanced the precocious alae phenotype of *hbl-1(ve18)* mutants: 76% of *hbl-1(ve18)* worms displayed either complete or gapped precocious alae in the L4 stage compared to 97% of *mir-52; hbl-1* double mutant worms (Table 2). Enhancement of the precocious phenotype of *hbl-1(ve18)* worms may reflect reduced activity of *hbl-1* itself, since *ve18* is a reduced function, not a null, allele [33]. Finally, *lin-14* functions to regulate the timing of both L1 versus L2 and L2 versus L3 cell fate decisions [35]. To analyze genetic interactions with *lin-14*, we used the temperature sensitive allele, *n179*. At 25°C, 34% of *lin-14(ts)* worms form complete precocious alae at the L3 to L4 transition compared to 76% of *mir-52; lin-14(n179)* worms (Table 2). Enhancement was not observed for the *lin-41, lin-42, or lin-28* phenotypes (Table 2). The enhancement of the precocious developmental timing defects observed in *mir-48(ve33), hbl-1(ve18)*, and *lin-14(n179ts)* mutant worms is consistent with a role for the *mir-51* family in the regulation of L2 versus L3 cell fate decisions.

### *mir-51* family members function upstream of *hbl-1*, but not *lin-28*, to suppress developmental timing defects in *let-7* family mutants

**Figure 1:**
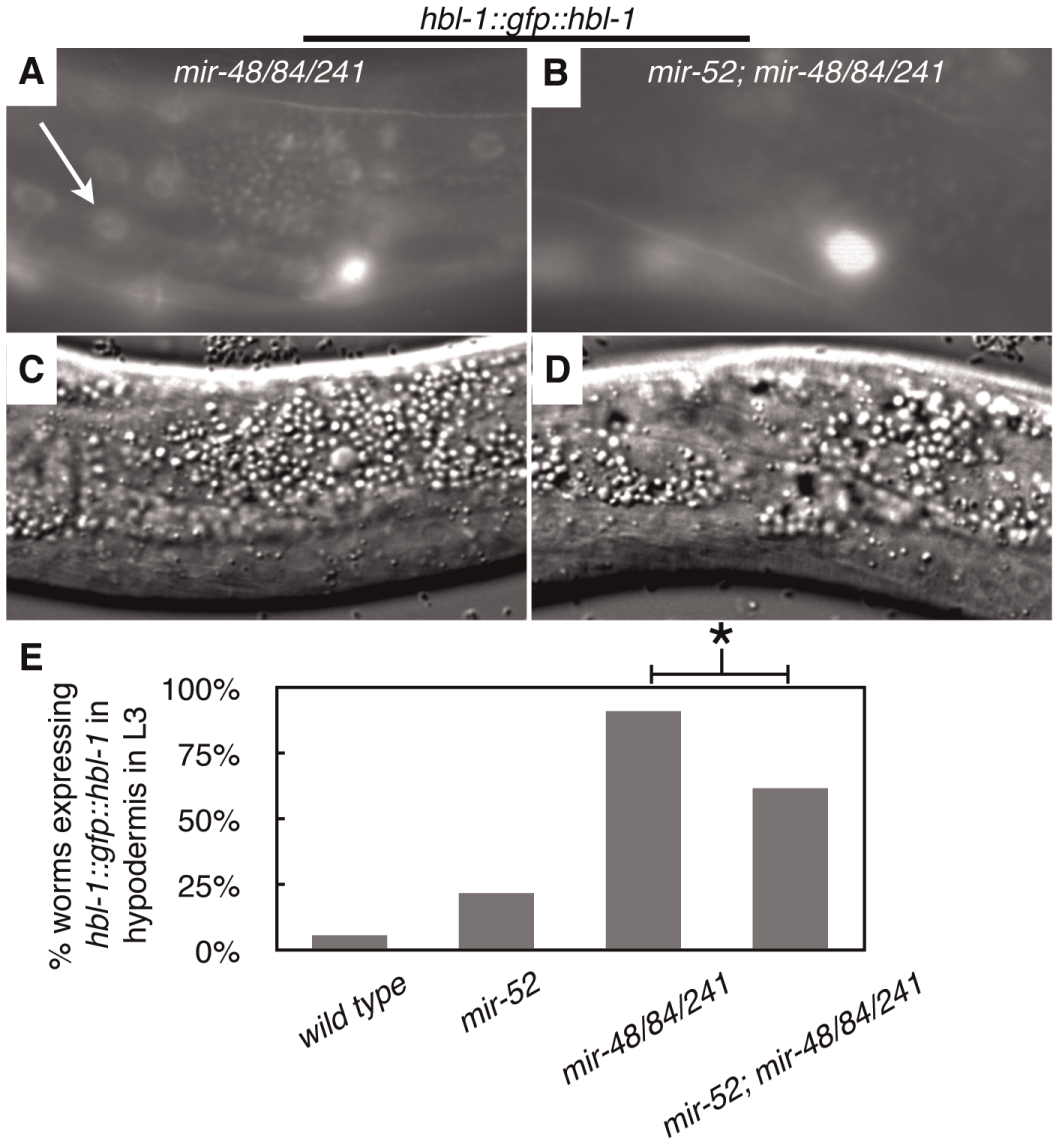
Loss of *mir-52* **suppresses** ***hbl-1*** **misregulation in** ***mir-48/84/241*** **mutants.** Representative fluorescent micrographs of *hbl-1::gfp::hbl-1* transgene expression in (A) *mir-48/84/241* and (B) *mir-52; mir-48/84/241* mutant worms in the L3 stage with corresponding DIC images (C and D, respectively). White arrow in A indicates a hyp7 nucleus. (E) Percentage of worms with *hbl-1::gfp::hbl-1* expression in hypodermis of L3 stage worms, n≥33 (range 33–37). * indicates significant difference (χ^2^, p<0.01).

Genetic interactions between *mir-52* and *let-7* family members as well as *hbl-1(ve18)* suggest that *mir-52* may act upstream of *hbl-1* to promote its activity. *hbl-1* is robustly expressed in the hypodermis during embryonic and early larval development and then is subsequently down-regulated through its 3′ UTR by the early L3 stage [33], [34], [36]. The down-regulation of *hbl-1* in the hypodermis requires the *let-7* family members, *mir-48, mir-84*, and *mir-241* [12]. We therefore determined whether the observed suppression of developmental timing defects in *mir-52; mir-48/84/241* reflects a suppression of *hbl-1* misregulation. Indeed, loss of *mir-52* partially suppressed the *hbl-1* misexpression phenotype of *mir-48/84/241* mutant worms: in 91% of *mir-48/84/241* worms *hbl-1::gfp::hbl-1* transgene expression remained high in L3, whereas only 62% of *mir-52; mir-48/84/241* displayed high *hbl-1::gfp::hbl-1* expression (Figure 1). This indicates that *mir-52* acts upstream of *hbl-1* expression in opposition to *let-7* family activity.

**Figure 2:**
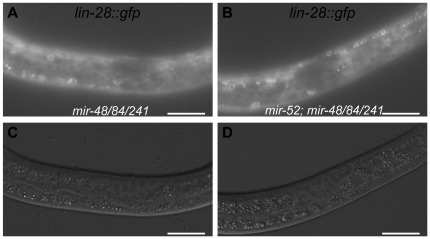
Loss of *mir-52* **does not result in increased expression of** ***lin-28::gfp::lin-28*** **.** (A, B) Representative fluorescent micrographs of *lin-28::gfp::lin-28* transgene expression at in (A) *mir-48/84/241* and (B) *mir-52; mir-48/84/241* worms in the L2 molt stage with corresponding DIC images, (C and D, respectively). Strains were scored for expression of *lin-28::gfp::lin-28* at the L2 molt (n = 17). No significant difference was observed between strains (χ^2^, p>0.05).

Like *hbl-1, lin-28* is also a critical regulator of L2 versus L3 cell fate decisions. We used a *lin-28::gfp::lin-28* transgene to determine whether *mir-52* suppression is the result of a misregulation of *lin-28*. However, no difference was observed in *lin-28::gfp::lin-28* expression between *mir-48/84/241* and *mir-52; mir-48/84/241* in L2 molt stage worms (Figure 2). Thus, misregulation of *lin-28* does not account for the observed suppression of developmental timing defects in *mir-52; mir-48/84/241* worms. Together, these data are consistent with the *mir-51* family functioning downstream or in parallel to *lin-28*, *lin-46* and the *let-7* family, but upstream of *hbl-1* to regulate the L2 versus L3 cell fate decisions.

### Loss of *mir-51* family members suppresses additional miRNA-dependent regulatory pathways in *C. elegans*

Genetic interactions with the developmental timing pathway may reflect a specific function for the *mir-51* family miRNAs in the regulation of targets in this pathway. Alternatively, these interactions may reflect a broader function for the *mir-51* family in the regulation of miRNA biogenesis or activity. For example, the developmental timing defects observed in *alg-1* or *ain-1* mutants [18], [20] are due to lower overall miRNA activity, including the *lin-4* and *let-7* family miRNAs, rather than a specific function in the developmental timing pathway. Therefore, we tested whether the *mir-51* family interacted with additional miRNA-regulated pathways by determining if loss of *mir-51* family members could suppress other miRNA mutant phenotypes that are distinct from developmental timing, including *lsy-6* regulation of neuronal asymmetry, *let-7* family regulation of vulva cell fate specification, *mir-240/786* regulation of defecation, *mir-35* family regulation of embryonic development and *mir-1* regulation of neuromuscular function.

#### lsy-6

**Figure 3:**
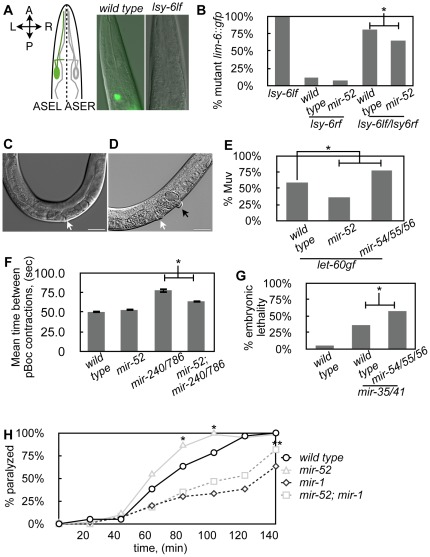
The *mir-51* family members, *mir-52* and *mir-54/55/56* , **function in multiple miRNA-dependent developmental pathways.** (A, B) *mir-52* suppresses ASEL specification defects of *lsy-6(rf)/lsy-6(lf)* worms. (A) Cartoon of *lim-6::gfp* expression in wild-type and *lsy-6(lf)* worms. A, anterior; P, posterior; L, left; R, right. (B) Worms of indicated genotypes were scored for *lim-6::gfp* expression in late larval and young adult stages, n≥169. * indicates significant difference (χ^2^, p<0.01). (C–E) Loss of *mir-52* partially suppresses, while loss of *mir-54/55/56* enhances, the multivulva (Muv) phenotype of *let-60gf* worms. (C) A wild type worm with one normal vulva, white arrow. (D) A *let-60gf* worm with one normal vulva, white arrow, and one ectopic vulva, black arrow. Bars represent 100 µm. (E) Synchronized L1 worms of the indicated genotype were allowed to develop at 25°C for 2–3 days and then scored as young adults for the Muv phenotype. n≥100. * indicates significant difference (χ^2^, p<0.01). (F) Loss of *mir-52* reduces the average defecation cycle time of *mir-240/786* mutant worms. Average time between consecutive pBoc contractions for n≥5 worms. * indicates significant difference (student's t-test, p<0.01). Error bars indicate SEM values. (G) Loss of *mir-54/55/56* enhances the embryonic lethality of *mir-35* through *41* mutant worms. L4 worms of the indicated genotypes were shifted to 25° and the next day embryos from these worms were collected. After 24 hours, unhatched embryos were counted to determine the percentage of embryonic lethality (n≥148). * indicates significant difference (χ^2^, p<0.01). (H) Loss of *mir-52* modestly suppresses the resistance to levamisole of *mir-1* worms. *mir-52* mutants show weakly enhanced sensitivity to levamisole. * indicates significant difference compared to wild type at the indicated time point (χ^2^, p<0.05). ** indicates significant difference compared to *mir-1* at the indicated time point (χ^2^, p<0.05).

The *lsy-6* miRNA specifies the ASEL cell fate through the down-regulation of its target, *cog-1* [14]. lsy-6 repression of *cog-1* is necessary for *lim-6::gfp* expression in the ASEL [14]. To achieve a genetic background with optimally compromised *lsy-6* activity, we used heterozygous worms that carry a loss of function allele, *ot149*, and a reduced function allele, *ot150*. 85% of these *lsy-6(ot149lf)/lsy-6(ot150rf)* heterozygous worms fail to express *lim-6::gfp* in the ASEL neuron compared to 100% of *lsy-6(ot149lf)* and 14% of *lsy-6(ot150rf)* worms (Figure 3B; [14]). Loss of *mir-52* partially suppressed mutant *lim-6::gfp* expression in *lsy-6(ot149lf)/lsy-6(ot150rf):* 85% of *lsy-6rf/lsy-6l*f worms displayed mutant *lim-6::gfp* expression compared to 61% of *mir-52; lsy-6rf/lsy-6lf* (Figure 3B).

#### *let-7* family regulation of vulva development

The *let-7* family miRNAs repress *let-60/RAS* in the regulation of vulva development [37]. Worms with a gain-of-function mutation in *let-60* display defects in cell fate specification, which often results in a ‘Muv’ phenotype with multiple vulva structures produced [38]. Overexpression of *let-7* family members partially suppresses the *let-60gf* Muv phenotype [37]. If the *mir-51* family opposes *let-7* activity in vulva development, as it did in the developmental timing pathway, then it would be expected that loss of *mir-51* family members should suppress the *let-60gf* Muv phenotype. This is observed in *mir-52;let-60gf* worms (Figure 3E). Interestingly, loss of *mir-54/55/56* enhanced the Muv phenotype of *let-60gf* (Figure 3E). This may reflect distinct activities of individual *mir-51* family members in the control of vulva development. Identification of *mir-51* family targets in the vulva specification pathway is required to elucidate the functions of individual *mir-51* family members.

#### mir-240/786

*mir-240/786* is necessary for the normal rhythmicity of the defecation motor program [10]. In wild type worms, a defecation motor program occurs every ∼50 seconds [39]. In *mir-240/786* mutant worms, the average defecation cycle time is increased [10]. We found that loss of *mir-52* significantly reduced the average defecation cycle time of *mir-240/786* worms (Figure 3F). Loss of *mir-54/55/56* had no effect on the mean defecation cycle time of *mir-240/786* worms (data not shown).

#### *mir-35* family

The *mir-35* family comprises eight miRNAs, *mir-35* through *mir-42*. These family members are redundantly required for embryonic development and mutants lacking *mir-35* through *mir-41* exhibit temperature sensitive embryonic lethality [13]. We found that loss of *mir-54/55/56* did not suppress the embryonic lethal phenotype of *mir-35/41* mutants, but rather significantly enhances this phenotype (Figure 3G).

#### mir-1

*mir-1* is necessary for normal neuromuscular function [40]. *mir-1* mutants display a resistance to levamisole-induced paralysis due to an increase in levels of its targets, UNC-29 and UNC-63 [40]. We found that loss of *mir-52* weakly suppressed the levamisole resistance phenotype of *mir-1* worms (Figure 3H). We found that after 140 minutes on 200 µM levamisole *mir-52*; *mir-1* worms were less resistant to levamisole compared to *mir-1* (Figure 3H). We also found that *mir-52* worms appeared to be slightly more sensitive to levamisole compared to wild type worms. Loss of *mir-54/55/56* had no effect on levamisole sensitivity of *mir-1* or wild-type worms (data not shown).

### Loss of *mir-51* family members does not broadly enhance miRNA biogenesis or activity

**Figure 4:**
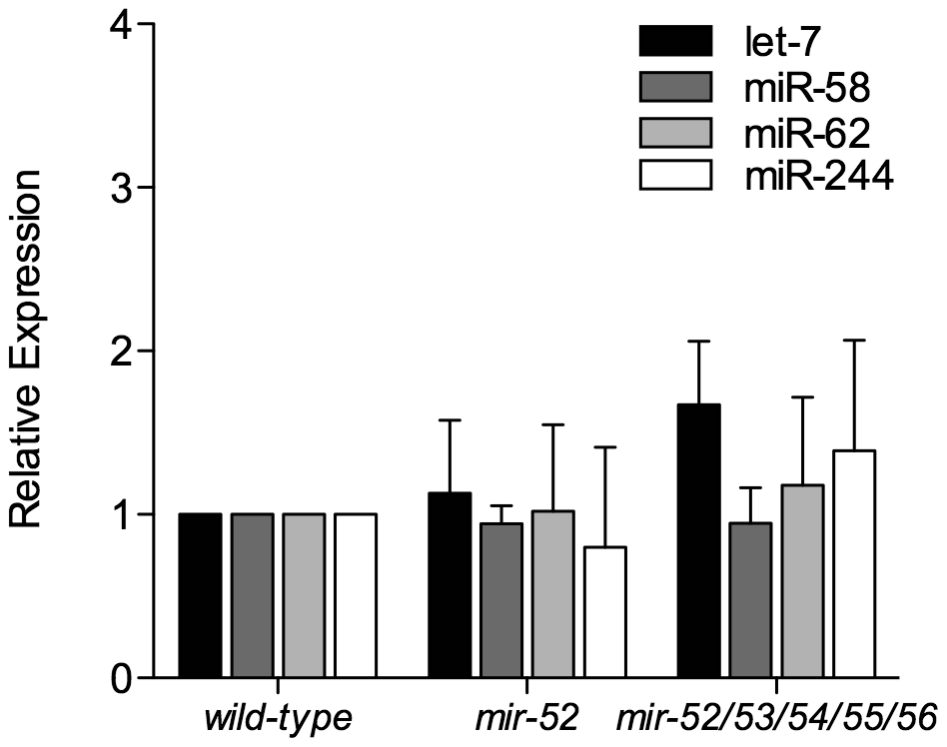
Levels of mature let-7, miR-58, miR-62, and miR-244 are unchanged in the absence of *mir-51* family members. Levels of mature miRNAs in wild type, *mir-52*, and *mir-52/53/54/55/56* mutant worms were measured and normalized to the average of two control RNAs, U18 and sn2343. The graph represents the level of mature miRNAs relative to wild type. Error bars represent the standard deviation (SD) between biological replicates. No differences in mature miRNA expression was observed (student′s t-test, p>0.24).

To account for the observation that the loss of *mir-52* suppressed multiple miRNA-dependent phenotypes, we proposed that *mir-52* may act to broadly regulate miRNA biogenesis or activity. To examine if the *mir-51* family regulates the miRNA pathway, we measured mature miRNA levels for a set of miRNAs that display various expression and biogenesis characteristics. We analyzed levels of the let-7 miRNA, a developmentally-regulated miRNA that functions in the developmental timing pathway in the hypodermis [5], miR-58, a highly abundant miRNA [26], miR-62, a miRtron that displays Drosha independent biogenesis [41], and miR-244, a miRNA that is expressed at lower levels primarily in hypodermal seam cells [29]. We found that the levels of these miRNAs are unchanged in *mir-52* mutants as well as in *mir-52/53/54/55/56* mutants (Figure 4). *mir-52/53/54/55/56* mutant worms display impenetrant embryonic lethality, slow growth, and mating defects [13], [25] indicating that *mir-51* family targets are sufficiently misregulated to result in severe, penetrant mutant phenotypes. However, no change in miRNA levels were detected for the four miRNAs analyzed. These results indicate that the observed suppression of developmental timing defects is not likely due to an increase in overall miRNA levels and that *mir-51* family miRNAs likely do not function broadly to regulate miRNA biogenesis.

**Figure 5:**
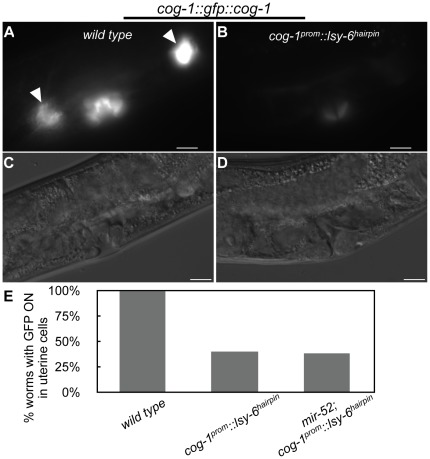
Loss of *mir-52* does not enhance the ability of ectopically-expressed *lsy-6* to regulate its target, *cog-1*. (A–E) Effect of *mir-52* on *lsy-6* mediated regulation of *cog-1::gfp::cog-1* expression. Representative fluorescent image of *cog-1::gfp::cog-1* transgene expression in (A) wild type worms and (B) worms with *cog-1::lsy-6* transgene with corresponding DIC images (C and D, respectively). White triangles point to uterine cells. Bars represent 10 µm. (E) Percentage of worms of given genotype with *cog-1::gfp* expression in either uterine cell, n≥20 (range 20–68). Worms were scored in mid-to-late L4 stage.

In order to determine if loss of *mir-52* can act to enhance miRNA activity, we analyzed the activity of ectopically expressed *lsy-6* in the repression of a *cog-1::gfp* reporter [14]. Ectopic expression of *lsy-6* under the control of the *cog-1* promoter allowed us to examine the activity of the lsy-6 miRNA in cells where it is normally not found, including uterine and vulva cells [14]. We found that in 60% of worms examined, ectopic expression of the *lsy-6* miRNA resulted in the down-regulation of *cog-1::gfp* in uterine cells (Figure 5). We found that loss of *mir-52* had no effect on the activity of ectopic *lsy-6* repression of *cog-1* (Figure 5). These data indicate that lsy-6 activity is not enhanced in the absence of *mir-52*, thereby suggesting that the *mir-51* family does not function broadly to regulate the activity of miRNAs.

## Discussion

The goal of this study was to define the mechanism whereby loss of *mir-51* family members can suppress the developmental timing defects of *alg-1* mutant worms. Our genetic evidence indicates that *mir-51* family members act early in the developmental timing pathway to regulate L2 versus L3 cell fate decisions. We observed that loss of the *mir-51* family member, *mir-52*, strongly suppressed the L2 stage reiteration phenotype of *mir-48/84/241* mutants and *lin-46* mutants. No significant suppression was observed with later acting genes in the developmental timing pathway, such as *let-7* and *puf-9*. Similarly, we observed genetic enhancement of precocious phenotypes due to mutations that result in omissions of early larval stage programs, like *lin-14* and *hbl-1*, but not mutations that result in omission of later larval stage programs, like *lin-41*. This suggests that the developmental timing pathway is the most sensitive to the loss of *mir-52* in the L2 stage.

In many species, including humans and flies, *mir-100, let-7,* and *lin-4* family members are located in a genomic cluster [42]-[44]. In flies, these three miRNAs are polycistronic and function together to regulate adult behaviors [42]. Although this clustered organization in the genome is not observed in worms, evidence herein supports a functional relationship between the *let-7* and *mir-51* family of miRNAs in the regulation of the developmental timing pathway.

Although *mir-51* family members interact with developmental timing genes, such as *let-7* family members and *lin-46, mir-51* family members are atypical developmental timing genes. First, unlike other developmental timing miRNAs, such as *lin-4* and *let-7*, *mir-51* family members do not display stage-specific expression but rather display nearly ubiquitous expression throughout development. In addition, loss of nearly all *mir-51* family members, which results in multiple defects including slow growth and embryonic lethality, did not result in developmental timing defects [13,25; Table 1 and Table 2]. We therefore propose that the *mir-51* family miRNAs are not themselves regulators of developmental timing decisions but that they likely act downstream in the execution phase of developmental programs.

Surprisingly, we found that *mir-51* family members displayed genetic interactions with multiple miRNA genes. These miRNAs function in diverse developmental and physiological processes, which include developmental timing, vulva fate specification, neuronal fate specification, defecation, and neuromuscular function. Loss of the *mir-51* family member, *mir-52*, partially suppressed the vulva cell fate defects of *let-60gf* mutants, the ASEL cell fate defects of *lsy-6* mutants, the defecation defects of *mir-240/786* mutants, and the levamisole resistance of *mir-1* mutants. These activities for the *mir-51* family may reflect the regulation of a single target that functions broadly in many pathways or the regulation of multiple targets that each function in distinct pathways. Our analysis of candidate targets failed to conclusively identify downstream *mir-51* family targets (data not shown).

One hypothesis to account for the observed suppression of multiple miRNA-regulated pathways is that the loss of an abundant miRNA such as miR-52 frees up miRNA-induced silencing complex (miRISC) so that it is available for binding by other miRNAs in a cell. Consistent with this model, the miR-51 family is both abundantly [26]–[28] and broadly expressed in tissues in which we observed a genetic interaction, including the hypodermis, the ASEL neuron, the vulva, the intestine, and muscle [25], [29], [45], [46]. In this model, excess miRNAs would compete for a limited pool of miRISC in wild-type worms in order to effectively repress their targets. In genetic backgrounds in which the activity of miRISC factors are reduced, such as in *alg-1* mutants, miRISC becomes limiting as evidenced by an increased amount of stem-loop miRNA precursors in both worms [18] and in human cells [47]. In human cells, overexpression of Argonaute-encoding genes results in an elevation of ectopically-expressed mature miRNAs [47]. However, it was not determined if endogenous miRNAs were elevated following Argonaute overexpression. In wild-type worms, precursor miRNAs are often detected in relatively low abundance [18], [48], [49]. These low levels of miRNA precursors that are detected may indicate either the competition for limited miRISC or the normal, steady state level of miRNA precursor in the biogenesis pathway. Although our genetic data are consistent with this limiting miRISC model, our molecular and transgene expression data are inconsistent with this model. First, it is expected that overall levels of all mature miRNAs would be elevated in mutants that lack the abundant miR-52 due to increased loading into miRISC. However, no such elevation in the mature miRNA levels was observed for the four miRNAs that were analyzed in the absence of *mir-52* or *mir-52/53/54/55/56* activities. In addition, loss of *mir-52* did not result in an enhancement of ectopic *lsy-6* activity in uterine cells as would be predicted by the limiting miRISC model. Future research will be directed at testing this model in order to determine if critical miRISC factors, such as Argonaute proteins, are limiting. Interestingly, in human cells and early *Xenopus* embryos, Argonaute protein levels are low and can be readily saturated by exogenous siRNAs [50], [51].

We found that the interactions with multiple miRNA-dependent pathways likely does not reflect the regulation of the miRNA pathway by the *mir-51* family. As described above, neither increased miRNA biogenesis nor increased miRNA activity was observed in *mir-51* family mutant backgrounds. Thus, *mir-51* family members likely do not function to regulate the core pathway required for all miRNA biogenesis or activity. However, it remains possible that *mir-51* family members may function to modulate miRNA activity in specific cells or for only a subset of miRNAs not included in our analysis.

We propose that the broad activity of *mir-51* family members reflects the repression of a target or multiple target mRNAs that act in distinct genetic pathways, possibly acting to fine tune, or buffer target protein levels. This broad activity described for the *mir-51* family is unlike that of previously described miRNAs in *C. elegans*. A direct target or targets of the *mir-51* family in the diverse development pathways described herein remain unknown. Of the 293 conserved targets predicted by Targetscan [52], [53], only 6 contain more than one binding site for the *mir-51* family and none of these 6 have more than two binding sites for the *mir-51* family. Because the *mir-51* family is broadly and abundantly expressed throughout development in *C. elegans* [25]–[28], multiple binding sites for the *mir-51* family within the 3′UTR of a gene would be expected to cause robust repression of that gene throughout development. Multiple sites or sites with high binding affinity may therefore be selected against during evolution. We speculate that the function of *mir-51* family members may be to weakly repress or fine-tune the protein levels for a large set of diverse downstream targets.

## Materials and Methods

### Nematode Methods

*C. elegans* were maintained using standard conditions. Strains used in this study are listed in Table S1. Worms were kept at 20°C unless otherwise indicated. All strains were constructed using standard genetic techniques [54]. PCR was used to confirm the genotype of strains that contained miRNA deletion alleles [17]. Fluorescence and differential interference contrast (DIC) microscopy were performed with a Nikon Eclipse 80i compound microscope equipped with a Photometrics CoolSNAP HQ2 monochrome digital camera and RS Image software (Roper Scientific) or with NIS Elements software (Nikon).

### Defecation assay

Worms were transferred to NGM plates at room temperature and allowed to equilibrate on plates for at least 5 minutes prior to scoring the time between consecutive pBoc contractions. 10 consecutive contractions was scored per worm.

### Levamisole sensitivity assay

Worms were transferred to NGM plates supplemented with 200 µM levamisole at room temperature, as done elsewhere [40]. Paralysis was assessed every 20 minutes over the course of 140 minutes by prodding worms with a wire. 20 worms were scored per strain in three independent assays.

### GFP reporter transgenes

The *syIs63* transgene was used to monitor *cog-1* repression in the presence of ectopic *lsy-6* expression driven from the *otEx1450* transgene array expressing *cog-1^prom^::lsy-6^hairpin^* as described in Johnston and Hobert (2003) [14]. This array was chromosomally integrated to generate *otIs193* (kindly provided by L. Cochella and O. Hobert).

### RNA Preparation

A mix of 1000 late L4 and L4 molt worms were collected for wild type, *mir-52*, and *mir-52/53/54/55/56* mutant worms. Total RNA was prepared using Trizol (Invitrogen) followed by isopropanol precipitation. RNA samples were DNase treated (DNA-free Kit, Ambion).

### qRT- PCR

10 ng of total RNA was used to analyze the levels of mature miRNAs with Applied Biosystems Taqman miRNA assays following the manufacturers protocol. Data was analyzed using 2^−ΔΔCt^ analysis [55] with the mean of U18 and sn2343 as reference. Two technical replicates were performed using two independently isolated total RNA samples for each strain. Each qPCR reaction was performed in triplicate. Student's t-tests were used to statistically compare the fold change of miRNA expression relative to wild type. No significant difference was identified between strains, p>0.24. RNA isolated from N2 worms was used to determine the % efficiency for each PCR assay, which was found to be >95% in each case.

## Supporting Information

Table S1 **Strains used in this study.**
(PDF)
