# Supplementary material for: The mir-51 Family of microRNAs Functions in Diverse Regulatory Pathways in Caenorhabditis elegans
Source: PLoS One. 2012 May 16;7(5):e37185. doi: 10.1371/journal.pone.0037185 (PMC3353893; doi:10.1371/journal.pone.0037185)
Supplement: Table S1 — Strains used in this study. (PDF) [file pone.0037185.s001.pdf]

Table S1. Strains used in this study	
**Strain**	Genotype
MT7626 *let-7ts*	*let-7(n2853) X*
MT14119 *mir-35 thru 41*	*mir-35/36/37/38/39/40/41(nDf50) II*
OH3645 *lsy-6lf*	*otIs114 I; lsy-6(ot149) V*
OH3646 *lsy-6rf*	*otIs114 I; lsy-6(ot150) V*
OH7310 *cog-1::gfp; cog-1::lsy-6*	*syIs63 otIs193 IV*
PS3662 *cog-1::gfp*	*syIs63 IV*
RF210 *mir-54/55/56; mir-35-41*	*mir-35/36/37/38/39/40/41(nDf50) II; mir-54/55/56(nDf58) X*
RF399 *mir-54/55/56*	*wIs78 IV; mir-54/55/56(nDf58) X*
RF415 *mir-54/55/56; mir- 48/84*	*mir-48(n4097) maIs105 V; mir-54/55/56(nDf58) mir-84(n4037) X*
RF440 *let-60; mir-54/55/56*	*let-60(ga89) IV; mir-54/55/56(nDf58) X*
RF442 *mir-54/55/56; let-7ts*	*let-7(n2853) mir-54/55/56(nDf58) X*
RF447 *mir-51; let-7ts*	*mir-51(n4473) IV; let-7(n2853) X*
RF448 *mir-52; let-7ts*	*mir-52(n4114) IV; let-7(n2853) X*
RF449 *mir-53; let-7ts*	*mir-53(n4113) IV; let-7(n2853) X*
RF451 *mir-51; mir-48/84*	*mir-51(n4473) IV; mir-48(n4097) maIs105 V; mir-84(n4037) X*
RF454 *mir-53; mir-48/84*	*mir-53(n4113) IV; mir-48(n4097) maIs105 V; mir-84(n4037) X*
RF462 *mir-52; let-60gf*	*mir-52(n4114) let-60(ga89) IV*
RF464 *mir-48/84/241; hbl-1::gfp::hbl-1*	*ctIs39 IV; mir-48 mir-241(nDf51) V; mir-84(n4037) X*
RF469 *mir-52; mir-48/84*	*mir-52(n4114) IV; mir-48(n4097) maIs105 V; mir-84(n4037) X*
RF473 *mir-52; hbl-1::gfp::hbl-1*	*mir-52(n4114) ctIs39 IV*
RF481 *wild type*	*wIs79 X*
RF483 *mir-53*	*mir-53(n4113) wIs78 IV*
RF486 *hbl-1::gfp::hbl-1*	*ctIs39 IV*
RF491 *mir-51*	*mir-51(n4473) wIs78 IV*
RF494 *mir-52; mir-48/84/241; hbl-1::gfp::hbl-1*	*mir-52(n4114) ctIs39 IV; mir-48 mir-241(nDf51) V; mir-84(n4037) X*
RF499 *mir-52*	*mir-52(n4114) IV; wIs79 X*
RF532 *mir-52; lsy-6rf*	*otIs114I; mir-52(n4114) IV; lsy-6(ot150) V*
RF534 *hbl-1*	*hbl-1(ve18) wIs79 X*
RF535 *mir-52; hbl-1*	*mir-52(n4114) IV; hbl-1(ve18) wIs79 X*
RF536 *lin-41*	*lin-41(ma104) I; wIs79 X*
RF537 *mir-52; lin-41*	*lin-41(ma104) I; mir-52(n4114) IV; wIs79 X*
RF538 *lin-42*	*lin-42(n1089) II; wIs79 X*
RF541 *mir-52; lin-42*	*lin-42(n1089) II; mir-52(n4114) IV; wIs79 X*
RF543 *mir-52; mir-240/786*	*mir-52(n4114) IV; mir-240 mir-786(n4541) X*
RF553 *mir-48/84/241*	*wIs78 IV; mir-48 mir-241(nDf51) V; mir-84(n4037) X*
RF554 *mir-48/84/241*	*mir-48 mir-241(nDf51) V; mir-84(n4037) wIs79 X*
RF555 *mir-51; mir-48/84/241*	*mir-51(n4473) wIs78 IV; mir-48 mir-241(nDf51) V; mir-84(n4037) X*

RF556 *mir-52; mir-48/84/241*	*mir-52(n4114) IV; mir-48 mir-241(nDf51) V; mir-84(n4037) wIs79 X*
RF557 *mir-53; mir-48/84/241*	*mir-53(n4113) wIs78 IV; mir-48 mir-241(nDf51) V; mir-84(n4037) X*
RF558 *mir-54/55/56; mir-48/84/241*	*wIs78 IV; mir-48 mir-241(nDf51) V; mir-84(n4037) mir-54/55/56(nDf58) X*
RF563 *lin-14*	*lin-14(n179) wIs79 X*
RF565 *lsy-6lf*	*otIs114I; lsy-6(ot149) wwIs5V*
RF568 *lin-46*	*lin-46(ma164) V;wIs79 X*
RF569 *mir-52; lin-46*	*mir-52(n4114) IV; lin-46(ma164) V; wIs79 X*
RF573 *mir-52; lin-28*	*lin-28(n719) I; mir-52(n4114) IV*
RF578 *mir-52; puf-9*	*mir-52(n4114) ; puf-9(ok1136) X*
RF583 *mir-52; mir-48(ve33)*	*mir-52(n4114) IV; mir-48(ve33) V*
RF588 *mir-52; lin-14*	*mir-52(n4114) IV; lin-14(n179) wIs79 X*
RF590 *mir-52; lsy-6lf*	*otIs114I; mir-52(n4114) IV; lsy-6(ot149) wwIs5 V*
RF61 *mir-240/786*	*mir-240 mir-786(n4541) X*
RF619 *mir-48/241*	*mir-48/241(nDf51) V; wIs79 X*
RF620 *mir-52; mir-48/241*	*mir-52(n4114) IV; mir-48/241(nDf51) wIs79 X*
RF622 *mir-52; cog-1::gfp; cog-1::lsy-6*	*mir-52(n4114) syIs63 otIs193 IV*
RF625 *mir-48/241; puf-9*	*mir-48 mir-241(nDf51) V; puf-9(ok1136) wIs79 X*
RF626 *mir-52; mir-48/241; puf-9*	*mir-52(n4114) IV; mir-48/241(nDf51) V; puf-9(ok1136) wIs79 X*
RF689 *mir-52; lin-28; lin-46; mir-48/84/241*	*lin-28(n719) I; mir-52(n4114) IV; lin-46(ma164) mir-48 mir-241(nDf51) V; mir-84(n4037) wIs79 X*
RF691 *mir-52; mir-48/84/241; lin-28::gfp*	*mir-52(n4114) IV; mir-48 mir-241(nDf51) V; mir-84(n4037) X; maIs108*
RF692 *mir-52/53/54/55/56*	*mir-52(n4100) mir-53(n4113) IV; mir-54/55/56(nDf58) X*
RF730 *mir-48/241; mjEx160[mir-54/55/56]*	*wIs78 IV; mir-48 mir-241(nDf51) V; mjEx160[mir-54/55/56]*
RF753 *mir-1*	*mir-1(n4102) I*
RF754 *mir-52; mir-1*	*mir-1(n4102) I; mir-52(n4114) IV*
RG490 *mir-48(ve33)*	*mir-48(ve33) V*
RG733 *wild type*	*wIs78[scm::gfp] IV*
SD551 *let-60gf*	*let-60(ga89) IV*
VC894 *puf-9*	*puf-9(ok1136) X*
VT1064 *mir-48/84*	*mir-48(n4097) maIs105 V; mir-84(n4037) X*
VT1102 *lin-28; lin-46; mir-48/84/241*	*lin-28(n719) I; lin-46(ma164) mir-48 mir-241(nDf51) V; mir-84(n4037) wIs79 X*
VT1138 *mir-48/84/241; lin-28::gfp*	*mir-48 mir-241(nDf51) V; mir-84(n4037) X; maIs108*
VT517 *lin-28*	*lin-28(n719) I*
VT808 *lin-28::gfp::lin-28*	*maIs808*
